# King's College Hospital

**Published:** 1903-10-17

**Authors:** 


### HOSPITAL ADMINISTRATION.

### CONSTRUCTION AND ECONOMICS.

### KING'S COLLEGE HOSPITAL.

DECISION TO REMOVE TO SOUTH LONDON.

Committee Appointed to Select the Site.

A special Court of the Corporation of King's College Hospital—president, vice-presidents, and governors—was called for the purpose of obtaining sanction for the removal of the hospital to a site in South London and held at King's College on Monday, the 12th inst.

The Hon. F. D. Smith, M P., was voted to the chair; and after the confirmation of the minutes of the last meeting of the Court the Secretary read a number of telegrams and letters from those unable to be present. H R.H. the Duke of Cambridge, the President, regretted his inability to attend the meeting, but said that he approved of the idea of moving the hospital to South London, and hoped his opinion would be communicated to the governors and committee. The Archbishop of Canterbury wrote that he had to go to Bristol, but was ready to support any plan approved by the principal and the signatories to the recently issued memorandum.

The Bishop of Exeter (Dr. Robertson, the late Principal of the College) wrote regretting that he would be unable to be present, adding:

"I may say that I fully share the feelings of those who would regret the removal of the hospital from the many local associations which gather round its present site.

"At the same time I am convinced that the removal, if a suitable site can be found, will be fraught with benefit both to the hospital and to its medical school, and to London generally.

"What is wanted now is a united front and resolution to put the removal successfully through. I hope it will be courageously begun and speedily accomplished."

Sir Albert Rollit's letter also expressed the view that the removal of the hospital was the proper policy to pursue.

On the other hand Miss Louisa Twining wrote that, as one of the first and oldest friends of the hospital, she most deeply regretted the proposed change; and her brother, Mr. Richard Twining, formerly Treasurer of the hospital, sent word that he had heard of the suggested removal with equal surprise and regret. Had his age and infirmities permitted him to be present it would have been to have expressed in very decided terms, as a subscriber to King's College Hospital from its commencement, his opposition to the removal of the hospital from its present site.

The Chairman then explained that it was necessary to call a meeting of governors, since it was impossible to deal with the land and buildings without an Act of Parliament. It was necessary for that Court to authorise measures so that a Private Bill might be introduced to carry out the scheme decided on, and for that resolution they must have a three-quarters majority. The legal aspect of the matter had been dealt with by the solicitors to the hospital, and the resolutions had been drawn up by them. He hoped the meeting would adopt a policy which had been approved by more than one committee of inquiry, by the medical staff, and by the Council of Management and the Council of the College.

Dr. Buzzard moved the first resolution: "That this Court approves of the proposal to remove King's College Hospital to a site in South London." It had been intended, he said, that the resolution should be proposed by Lord Lister and seconded by the Bishop of Exeter, but unfortunately neither was able to be present; and as the matter had only been put into his hands as he came into the theatre, he must apologise if he failed to support it in the way the intended mover would no doubt have done. However, they all had in their hands the letter signed by those interested in the question, which in a very graphic and lucid manner conveyed the reasons for the suggested removal. There was, therefore, no need for him to go through all the arguments again. But he was old enough to have been a resident medical officer in the original King's College Hospital 50 years ago, and so was in a position that he could, not only speak from knowledge of the extraordinary difference in the character of the neighbourhood, but was also filled with sentiment regarding the prospect of moving to another site. He had not the least doubt as to the necessity for removal, and he thought the committee were not exceeding their duty in urging the governors to accede to the proposals, which had received the most careful, laborious, and studied consideration. The altered character of the neighbourhood, due to the improvements brought about first by the removal of the Law Courts to that locality and more recently by the changes now going forward in the Strand, had entirely altered the conditions under which King's College Hospital was originally founded. Then it was an exceedingly poor neighbourhood; now many of the poor people had gone away and more and more were seeking the suburbs. All the great hospitals were inconveniently crowded into a very small area. There was very little distance between King's and Charing Cross, or between King's and Bartholomew's, between Bart's and Guy's, or Guy's and the London, between Charing Cross and the Westminster, St. George's and St. Thomas's. Therefore, in taking the step of moving King's College Hospital they would be performing a great public duty. In defiance of their own sentiment, and facing very great difficulties, yet they felt it to be for the public good to move out into the suburbs to which the poorer population was drifting every hour. That hospital was peculiar in another respect in that it was founded, not only to supply the wants of the poor in the neighbourhood of the Strand, but as a field and ground of study for the medical school of King's College. The building was originally an old workhouse infirmary, unwholesome to a degree that would be considered terrible nowadays, but which subserved a useful purpose. That gave way to the present buildings; an important change with which they were greatly pleased. Time passed on, and the requirements of the hospital and of the medical school enormously increased. He could remember, for instance, when scarcely any instruments of observation were employed in the examination of the sick; when the temperature of the patient was tested by the hand of the physician; before the introduction of the thermometer; and before the days of the ophthalmoscope and the laryngoscope. All this sort of thing meant an enormous increase in the accommodation required, and their accommodation was proving increasingly insufficient. Medicine was a science now; formerly it was an art, and its practice largely empirical; and in surgery the change and contrast to what formerly occurred was extraordinarily great. In the existing hospital they could not keep pace with the change. It was too small for the work, and it was incapable of extension. Only by changing the site could they hope to keep abreast of modern requirements, both as regards the wholesomeness of the hospital itself and the needs of the medical school. The education of the medical man concerned the whole community, and it must never be forgotten that the well-being of the medical school was as important as the accommodation of the poor patients themselves. In both these respects could a case be made out for removal to another site. The deficiency of accommodation was acting detrimentally on the school, for students would not come where the requirements of the progress of medical science were unfulfilled. He would again refer them to the letter that had been issued, for in it were summed up all the arguments on the question at issue.

Mr. Charles Awdry, the Treasurer, seconded the resolution. He had, he said, considered very carefully what was their duty in this matter. As Treasurer, and because it was clear it would involve the spending of a great deal of money, he had most reluctantly been led to the conclusion that it was their duty to the poor as well as to the school to move the hospital from its present site to one more in the centre of the labouring population, where it would more fully meet the design of the original founders. There was no occasion for him to add much to what Dr. Buzzard had said, and to what was contained in the circular issued to them. They had but to consider the ground space to see that it was not possible to bring the hospital up to the proper standard of efficiency so long as they had only one acre available, and that was the conclusion to which they had again and again been forced. The present view of hospital construction was that only about 50 patients should go to the acre; and if that were accepted it was clear that by no stretch in any direction that was available could their accommodation be made even approximately adequate. The moving of the poor population from their neighbourhood was of course their main reason for moving—(hear, hear)—but they must remember that the medical school was an essential part of the foundation scheme of that hospital, and also that they could not do their best for their poor patients unless they had a medical school. (Applause.) Their great professional men gladly gave their services in teaching and in attendance, and without the school their patients could not possibly get the best attention. It could not be left out of consideration.

Mr. S. F. Wilde, who said he was one of the oldest subscribers to the institution, was sorry to have heard the talk about removal, but had almost been prepared to vote for it until he saw the letter in the *Times* signed by the chairman (Lord Dillon) and nine other gentlemen opposing it. He thought ten was a large proportion of the committee, and that put a different complexion on the matter. Surely they ought to have been told in the circular they were considering that the committee was not unanimous. As to the treasurer's statement about not having more than fifty patients to the acre, why no hospital in London came up to that standard. According to that, every hospital in London ought to be rebuilt! He agreed that the population of the neighbourhood had altered, but they still had a good many poor people there. From the report of the hospital he saw that they had last year no fewer than 7,482 emergency cases and 370 maternity cases. Surely that proved that the hospital was of great use? Charing Cross Hospital was not in a poor locality, yet a great deal was being spent on it; and so with St. George's. He looked upon this proposal as a step in the direction of separating the hospital from King's College, toward which there seemed lately to have been some tendency, to judge by the fact that no collection for the hospital had been taken in the college chapel since 1898. No doubt there was a good deal to be said in favour of moving, but this was not an opportune time—so many other institutions were just now begging for money. He should vote against the resolution.

Lieut.-Col. Clifford Probyn said he agreed with much the previous speaker had said, and to put the matter in order he would move an amendment. He wanted to conserve the hospital for that district. The district was undergoing a great change, and until it was more settled it was not right that the resolution should be passed. He would move that the matter be further considered and that it be postponed for two years. During that time the poor would be re-established in the model dwellings the County Council would build; the streets now being made would be built on and occupied. He therefore moved as an amendment to the resolution that the further consideration of the motion before the meeting be postponed for two years.

Mr. Wilde seconded.

Mr. H. S. Collins said it seemed to him waste of money to allow the people at Charing Cross Hospital to purchase ground and extend their building. Some steps should have been taken by the committee of King's to bring the two together. They could not lose sight of local sentiment. He himself had recently given £1,000 purely out of local sentiment, and that would all cease if they went to Camberwell.

The Rev. Dr. Headlam, Principal of King's College, said with regard to those nine members of the committee whose names were appended to Lord Dillon's letter, in many cases they did not resign because they were opposed to the principle of moving, but because they were frightened by the difficulties and expense. And in the case of one of them—Canon Ainger—he had heard that afternoon that he was surprised to see his name with the others and was not conscious that he had resigned. Of course there would be difficulties but if the step were a right one they ought to face them, and for the expense they must put the matter before the public. Reference had been made to Charing Cross Hospital but he thought the additions being made to that hospital were a further reason why King's should remove. Certainly there was room for a hospital in that neighbourhood, but not for two. If ten or twelve years ago it had been possible to make additions to King's, then probably Charing Cross would have had to move now. As to their medical students, they had, under present conditions, to meet cases necessary for their training. As to efficiency, it had been said that no hospital came up to the ideal of what was required, but if he could take the governors round the premises and show them the bad sanitary arrangements of some of the wards, and the dark poky rooms of the out-patients' department where operations had sometimes to be performed, they would have no doubt as to the inefficiency of the present building. There was only a certain number of hospitals that the charitable public was able to support, and the concentration of them all into a limited area did not facilitate the getting of that support. They could not meet the needs of London without taking the hospital from the centre and placing it nearer the circumference. He hoped they would be able to place it where it was wanted, and to erect a hospital worthy of their record and up to the standard of modern medicine and surgery. (Applause.)

Mr. C. E. Layton said that, as one of the members of the committee who had resigned, he was bound by the agreement come to, to take no action in the matter; but in view of the statement of the Principal regarding Canon Ainger's resignation he felt bound to ask for some explanation. Were they to understand that his signature was not appended to the letter?

Colonel Fellows explained that the matter had been discussed informally and that the Canon wished them to look upon him in the light of one ready to return to the fold.

(Laughter.) He did not wish to persist in his opposition to the majority.

Professor MacHardy said that some time ago he was convinced of the necessity for the removal now proposed, and after working at it for a considerable time was very gratified to find that their treasurer saw that that course was inevitable in the interests of the hospital itself, in the interests of the neighbourhood, in the interests of the charity, and in the interests of the medical school. The opportunity offered now as it might not two years hence. That institution had a history second to none, and no other hospital in the world could point to having been so much the means of introducing to the world all that was associated with the name of Lister. (Applause.) He was far from being destitute of sentiment, and he felt the less modesty in addressing them because they knew the number of years he had worked at the hospital and the amount of time and thought and work he had given to hospital construction and such matters. All the world over, for 30 years he had been studying the subject of hospital construction, hospital needs, and hospital advancement. There was one hospital which 30 years ago was a pioneer and was put up regardless of everything but efficiency, and intended to be an ideal institution. He need not tell those who studied hospital construction and requirements that he referred to the Johns Hopkins Hospital in Baltimore. There they put up a hospital not equal to anything that was, but superior to anything that had been. That should be their idea also. He thought they ought to make sure in their resolution—in justice to the charity with which they were connected—that in moving they should move to an adequate and suitable site, with no idea of it being a pinched institution because other hospitals were crowded. But, on the other hand, they should look round and avoid the extravagance they saw in some places. To attract medical students a hospital should be a large one. Their site should be ample for at least 600 beds. They should start everything on a scale that should be capable of developing, without pinching, to 600 beds. They proposed to go into the south of London. The poverty of the East End was fashionable, but it was nothing compared with the poverty of South London. He spoke well knowing what he was talking about, for he had been working in South London over 30 years. They could get an adequate and suitable site there, for he held in his hand offers of three different sites of from 11 to 16 and 20 acres, which they could get at prices of from £3,000 to £4,500 per acre. If they did not secure one of these sites they would quickly be picked up, for there was no way in which suburban property paid better than cut up for labouring people's cottages. He supported the resolution most heartily, but he should like it to read, "That this Court approves of the proposal to remove King's College Hospital to an adequate and suitable site in South London." He thought they should ensure this or at any rate indicate to those who would carry it out that they desired the site to be adequate and suitable, and the hospital to be an ideal one. (Applause.)

The Chairman suggested that the proposed words would be better added to the next resolution, and Colonel Probyn's amendment in favour of delay was then put and lost by a large majority, only six hands being held up for it. The resolution was then carried, the voting being the same.

Dr. Ferrier then moved, "That this Court accepts the report of the Sites Committee and authorises the Joint Committee appointed by the Council of King's College and the Committee of Management of the hospital to act for them in this matter and to purchase a site." They must have a committee to carry the first resolution into effect, and they could not do better than appoint those committees whose members had already given much time and attention to the matter.

Mr. Watson Cheyne seconded.

The Chairman read the report of the Sites Committee, which was as follows:—"The Committee desires to report that there are desirable sites available and that negotiations are proceeding. The Committee would prefer to leave the ultimate selection to the vote of the General Committee, when, after the sanction of the Governors has been obtained to the removal, the final arrangements for acquisition can be entered into."

Mr. McHardy moved to substitute for the words "to purchase a site" the words "to acquire a suitable and adequate site." At any rate, they ought not to have less than 10 acres; and since they would require a lot of money for building, they might not want to sink money on immediate purchase. They might build on lease with power to acquire the freehold.

The amendment was agreed to, and the resolution unanimously adopted.

Lieutenant-Colonel Fellows moved:—"That this Court authorises the Council of King's College, and the Committee of Management, to apply to Parliament for an Act to contain any powers necessary to carry out the proposed removal of the hospital, and power to sell, mortgage, lease, or otherwise dispose of the present site thereof, according to the terms of the memorandum appended hereto, with any other powers the Council may deem necessary, and subject to such alterations and additions as Parliament may make therein."

Mr. F. S. Wilde seconded, and it was carried *nem. con.*

A vote of thanks to the Chairman terminated the proceedings.

The memorandum of the terms of the Act of Parliament to be applied for is as follows:—

The Act shall provide that—

1. Such powers shall be given as shall be necessary to enable the Corporation, acting by their Committee of Management, with the approval of the Council of King's College, London, to carry out the proposed removal of the hospital.

2. (*a*) It shall be lawful for the Corporation, acting by their Committee of Management, with the approval of the Council of King's College, London, to sell, mortgage, lease, grant tenancies, or dispose in any other manner of the present site of King's College Hospital or any part thereof, and to convey, lease, or otherwise grant, demise, or dispose of the same, freed and discharged from any restrictions (statutory or otherwise) which may affect the same by reason of any part or parts thereof having been used or set apart for purposes of interment or having been consecrated, or being subject to any restriction or trust for the use of the same for the purposes of a hospital only, and to effect any such purposes to get in outstanding estates or interests in the land now occupied by the present hospital, and any deeds, instruments, or writings required to give effect to the purposes aforesaid shall be sealed, executed, or signed in the manner provided by the King's College Hospital Act, 1851.

2. (*b*) For any such purposes as aforesaid, to enter into any agreement for the sale, lease, or disposal in any other manner of the said present site, or any part thereof, on such terms of payment, whether by a sum in gross or by annual sums terminable or otherwise, or by way of rent and charged on, or reserved, or issuing out of the said site or otherwise, or partly in one way and partly in the other, and generally on and subject to such terms and conditions as may be agreed, and to alter, pull down, or destroy the present buildings of the said hospital, or any part or parts thereof, or suffer, or enter into contract for the same to be done, notwithstanding that any part or parts thereof may have been consecrated, and without the necessity of any faculty for any such purpose.

3. It shall be lawful for the said Committee of Management to cause or allow to be removed any human remains buried in any part of the present site of King's College Hospital, under such superintendence and subject to such approval and in such manner and to such places and at such expense, and subject to such reference in case of dispute as may be proper or be required, and without the necessity of any faculty for any such purpose; and also to remove all monuments and tombstones to such places and subject to such consent (if any) as may be required.

4. All the costs, charges, and expenses, preliminary to, and of and incidental to the preparing, applying for, and passing of the Act shall be paid out of the general funds of the hospital.

Following is the text of the circular letter sent to the Governors and referred to by the various speakers:—

Dear Sir,—As you are probably aware, a meeting of the Governors of King's College Hospital has been summoned for Monday, October 12th, at 4 p.m., at King's College. At that meeting the Governors will be asked to sanction the removal of the hospital from its present site to one in South London. This proposal is put forward at the initiation both of the Council of King's College and the Committee of the Hospital; it is supported by the unanimous opinion of the medical staff; it has not, you will well believe, been arrived at without very great thought and deliberation, and we feel that it is our duty to put before you the reasons which have induced us to recommend so serious and important a change.

The first point we would ask you to consider is the condition of the existing buildings of the hospital. While the wards are for the most part cheerful, light, airy, and convenient, in most other respects the hospital is not up to the standard of accommodation and sanitary sufficiency which is demanded, and rightly demanded, for a modern hospital. To quote from a memorandum of the medical staff:—"The present hospital building is not only inadequate in accommodation, but is also obsolete in design, insanitary in arrangement, and wholly behind the hygienic requirements of the day"; and, again, a committee of the same body wrote:—"The out-patients', the casualty, the educational, the resident, the students', and the teaching quarters are inadequate, inconvenient, out of date, and unattractive to all those for whose good they should exist." A visit to the hospital on the part of anyone acquainted with hospital arrangements will certainly corroborate these statements.

For a long time it was the hope of the committee to remedy some or all of these defects, and to raise money for that purpose by an appeal to the public; but on investigating the matter it became apparent that any adequate extension or improvement on the existing site would be very difficult, if not absolutely impossible. To quote from the same memorandum:—"The method of construction and the extremely narrow limits of the site upon which it is built are such that it is incapable either of any adequate extension, or of any remodelling that would bring it up to the standards of modern science and hygiene.

And a joint committee of inquiry appointed by the Council and Committee report:—"It is not apparent whether within the existing limits of the site upon which the hospital is built any adequate extension or any remodelling, such as to bring the hospital up to the standards of modern science and hygiene, is possible. This possibility has certainly been diminished by the inability to reacquire a plot of land on the south-west side of the hospital front, which, under very severe financial stress, was sold some years ago, before the question of additions to the present buildings had been brought before the Committee. As a result, the ground available for extension is limited in area and inconvenient in shape, while rights of light vested in the present owners of the alienated ground further restrict the possibility of making full use of the ground still uncovered by buildings."

No doubt means might be found for getting over some at any rate of the lesser defects, but it is very doubtful whether it would be either wise or right to spend £60,000 or £100,000 in patching up a building which must still be inadequate. Moreover, a further series of considerations, partly general in their scope, partly special, suggest that the hospital is not to so great an extent as formerly needed in its present site, and that therefore it would be very much wiser to transfer it to some locality where it would be more widely useful.

In the first place, the district in which it is situated has very greatly changed. When King's College Hospital was founded some 60 years ago, the locality afforded an admirable site for a hospital, which met a definite local need. North of the Strand was one of the poorest and most thickly populated districts in London. The change began in the clearance of the site of the present Law Courts and the streets immediately surrounding them. The result of that change was shown in a very large falling off in the number of out-patients that attended the hospital, a falling off which has never been made up.

The process then begun has been continued in the clearances connected with the Strand improvements, which have completely transformed the district. A few poor streets have been left, a certain number of the families cleared out have been replaced in workmen's dwellings let at a higher rental, but the greater number of the new sites will be used for office accommodation of various kinds, and the poorer inhabitants of the district have moved either south of the Thames or north of Holborn; in this latter case they come within the range of other hospitals. The recent County Council improvements will effect a permanent reduction of the population to the extent of at least 4,250 poor residents, and other changes of a similar character are taking place.

The result of this is that the hospital has ceased to have any special claims to support as a local charity. A large proportion both of the out-patients and the in-patients of the hospital now come from distant parts of London. On a single day, out of 213 in-patients only 10 came from the large parish of St. Clement Danes, in which the hospital is situated; 136 came from the County Council area, of these latter only 42 from within two miles of the site; and 67 from the provinces. Out of 211 out-patients on one day only 29 came from the hospital parish, and 174 came from more or less remote parishes in the County Council area, including more than 100 (nearly half the whole number) from South London.

But these particular figures are only a single illustration of what is true of Central London generally. By far the larger number of London hospitals are collected within a comparatively small area which is continually decreasing in population, ranging from St. Bartholomew's in the east to St. George's Hospital in the west, and the area is oversupplied with hospital accommodation. Whether or no other hospitals are well advised in remaining where they are is for them to decide, but the fact that Charing Cross Hospital has not only decided to remain, but has found itself able to increase its buildings, is an additional reason for other hospitals in the neighbourhood to consider the question carefully. For any population remaining in the immediate vicinity there is ample accommodation in St. Bartholomew's, Charing Cross, and the Royal Free Hospital. Although, therefore, for other reasons King's College Hospital might continue for a time to serve the needs of a distant *clientèle*, the need of it as a local charity has ceased to exist. On the other hand, there is an urgent popular demand for a large general hospital in South London, where there are great and growing masses of population three or four miles from any hospital. This is a fact which is recognised by the committee of the large hospital funds, and the hospital transferred to such a locality will fulfil a very important charitable demand.

So far we have only touched on the work of the hospital as a charity, but connected with this and of equal importance is its work as a medical school. The hospital was originally founded, not only as a charity, but also as a medical school for King's College, and, compared with other London hospitals, its importance has depended, more than perhaps has theirs, on the reputation of its medical school; but on the present site, and under the new condition of affairs, it will be very difficult if not impossible for the medical school to preserve its existence. This is a matter which concerns the council perhaps more than the governors, and need hardly be dwelt on now at great length, but the matter has been carefully investigated by both the medical staff and the council, and it is clear that the great decrease in the number of out-patients makes it impossible for students to obtain in such a neighbourhood the requisite experience. For instance, the number of maternity cases has fallen in ten years from 684 to 389. The decline of the medical school must quickly react upon the hospital. At present the value of the hospital as a charity depends largely upon the reputation of the medical school. The patients are largely sent to the physicians and surgeons by general practitioners in London and the country, and the out-patients are attracted by the excellence of the special departments. Any further decline, therefore, in the medical school will still further diminish the value of the hospital. How far in other cases the value of a hospital depends upon its medical school it is difficult to say, but in the case of King's College Hospital the dependence is undoubted.

The above reasons have seemed to us amply to justify the proposal which is now put before you to move the hospital to another site. The scheme in its main outline is as follows:—

1. The acquisition of a good site in South London. There is a very large area extending from Wandsworth, through Balham, Brixton, Stockwell, Camberwell, and Peckham to New Cross, which particularly needs the advantages implied in a large general hospital.

2. As experience shows, it is often more difficult to obtain money for the support than for the building of a hospital. It is proposed, therefore, that the value of the present site and buildings should be utilised as endowment. The advice of able men of business is that the existing buildings are capable of being utilised for other purposes without material alteration, and that it would be wiser if possible to let them on a lease. It is calculated that in that way a sum of possibly about £5,000 a year could be obtained for the future endowment of the hospital.

3. It is recognised that, in spite of every effort that is made, the annual subscriptions to the different hospitals and hospital funds could not be increased so as to enable a new hospital to be built in the large and populous district of South London to which we have referred without damage to existing hospitals, most of which already have difficulty in making both ends meet. It is therefore a wise and right thing to transfer a hospital from a site where there is little demand for it to a district in which there is a great and growing poor population, so that the endowments, the income, the prestige, and the eminent medical staff may be of far greater public utility, and may fulfil a far wider charitable function.

4. As far as regards the medical school, there will no doubt be some disadvantage in the distance of the hospital from the college. But alterations in the arrangements of the London medical schools which are probable in the future will, we hope, largely counteract that. The lecturers and professors of the college will, as a result of these changes, have a larger sphere of usefulness before them, and the erection of a new hospital with ample accommodation for students, built in accordance with the latest requirements of medical science, will, we may reasonably expect, attract large numbers to a school which has in past years played a leading part in the medical education and research of London.

It is not necessary at present to go into further detail. So soon as the consent of the governors is obtained the matter will be pushed forward with the greatest vigour. A sum of £300,000 will have to be collected, towards which £20,000 has been promised, and there are other promises of support. In putting this before the governors we are quite conscious of the attachment and affection which many must necessarily feel for the old site. No persons feel this more than the committee and staff of the hospital, both medical and nursing, but they also feel, and feel rightly, that all such considerations should make way for the public good, and that they cannot be allowed to outweigh the very strong arguments on the other side. We believe that the change, which has been carefully and fully discussed, is a right one, and will prove beneficial for the community, for the increased usefulness of the hospital, and for the good of the medical school, and therefore, in spite of certain difficulties and disadvantages, of which we are fully conscious, we earnestly put the proposal before you for your acceptance.

We have the honour to be yours faithfully,

| | |
|---|---|
| Salisbury. | Raymond Crawfurd. |
| A. F. London. | C. F. Fellows. |
| Edgar Alban. | David Ferrier. |
| Lister. | A. C. Headlam. |
| Glenesk. | Edwd Liveing, M.D. |
| R. C. Parsons. | A. H Aylmer Morton. |
| W. F. D. Smith. | Edwd. E Micholls. |
| John A. Cockburn. | Thomas Parker. |
| C. J. Lyall. | Nestor Tirard. |
| W. H. Preece. | H. H. Twining. |
| Owen Roberts. | Arthur Whitfield. |
| C. Awdry. | Jno. Willson. |
| T. Buzzard, M.D. | A. F. Yarrow. |

